# Unpacking the impact of teacher support on EAP-related perceived academic achievement in EMI context: the mediating role of self-efficacy and academic engagement

**DOI:** 10.3389/fpsyg.2026.1830742

**Published:** 2026-05-11

**Authors:** Yanping Yu, Yanling Feng, Dan Liu, Lei Liu

**Affiliations:** School of International Studies, Zhengzhou University, Zhengzhou, China

**Keywords:** academic engagement, English for academic purposes, perceived academic achievement, self-efficacy, sequential mediation, teacher support

## Abstract

**Introduction:**

This study examines how teacher support influences the EAP-related perceived academic achievement of engineering students in Sino-foreign cooperative “4+0” dual-degree programs. Grounded in Social Cognitive Theory, we propose that teacher support affects perceived academic achievement both directly and indirectly through self-efficacy and academic engagement, in a cross-cultural higher education context.

**Methods:**

A cross-sectional survey was administered to 500 first- and second-year undergraduate students at a comprehensive university in China. Structural equation modeling was used to analyze the hypothesized relationships among teacher support, self-efficacy, academic engagement, and EAP-related perceived academic achievement.

**Results:**

Teacher support showed a significant positive association with perceived academic achievement. Self-efficacy and academic engagement independently mediated this relationship. Moreover, a significant sequential mediation pathway was identified, in which teacher support is associated with self-efficacy, which in turn is linked to academic engagement, and ultimately to higher EAP-related perceived academic achievement.

**Discussion:**

The results highlight the vital role of teacher support in strengthening students’ motivation and engagement, thereby fostering their subjective academic success in an internationalized educational setting. Practical implications for teaching in similar cooperative programs are discussed, emphasizing the need for holistic instructional support to enhance student development.

## 1 Introduction

Over the past decades, internationalization has become a core strategy for higher education reform and a prominent global trend among universities (Tran et al., 2023). As China continues to accelerate the internationalization of its higher education system, universities—particularly Sino-foreign cooperative institutions—have placed great emphasis on the cultivation of talents with global competence (Zhuang et al., 2024). The “4 + 0” dual-degree program, a well-established model of Sino-foreign cooperation in higher education, allows students to complete their entire undergraduate education domestically while earning degrees from both Chinese and foreign institutions. According to the latest statistics from the Ministry of Education of China, the “4 + 0” model currently accounts for approximately 17% of all Sino-foreign cooperative programs and institutions (Ministry of Education of China, 2025), establishing itself as a significant configuration of internationalized higher education in the country.

The unique research value of the “4 + 0” model lies in its sustained requirement for academic adaptation, which differs significantly from “2 + 2” or “3 + 1” models. In this context, disciplinary courses are primarily delivered through English-medium instruction (EMI), referring to the use of English to teach non-linguistic academic subjects (Macaro et al., 2018). Whereas other models treat English for Academic Purposes (EAP) as short-term preparation for overseas study, the “4 + 0” model requires four-year EMI immersion in a non-English-speaking environment. It integrates international academic standards with local pedagogical practices and creates a unique cross-cultural and language-mediated academic environment. Consequently, students face sustained linguistic and academic demands, as well as “at-home” cross-cultural challenges, making the role of continuous teacher support particularly critical—yet under-researched—compared to models involving physical relocation.

Within this internationalized educational context, EAP courses play a foundational role in preparing students for participation in English-medium disciplinary learning. In many Sino-foreign cooperative programs, EAP instructors function not only as language teachers but also as academic mediators who help students bridge the gap between their previous English learning experiences and the expectations of internationalized academic discourse. Through various forms of instructional and interpersonal support, EAP teachers assist students in developing the linguistic competence, academic strategies, and confidence required for successful engagement in English-medium disciplinary study. For students in “4 + 0” programs, therefore, EAP learning is not merely about completing a language course; rather, it serves as a critical gateway to accessing and understanding other subject courses delivered in English. Therefore, in EMI context, academic learning is largely realized through EAP-related competencies, as students rely on these skills to access, process, and demonstrate disciplinary knowledge.

However, the transition from general English learning to EAP and EMI contexts presents considerable challenges. Students must adapt to cognitively demanding academic discourse while simultaneously processing complex disciplinary knowledge in a second language. This shift requires not only linguistic competence but also the ability to navigate unfamiliar academic conventions and learning expectations. In addition, high-stakes modular assessments commonly adopted in internationalized programs further intensify academic pressure and emotional strain. Under such demanding academic conditions, how students perceive their academic progress and learning success becomes particularly important.

In high-pressure learning environments, perceived academic achievement (PAA) serves as a crucial indicator of students’ academic adaptation. PAA, a term treated here as synonymous with perceived academic performance following prior conceptualizations (e.g., Izaguirre et al., 2023), generally refers to students’ cognitive perception of their academic outcomes, including their attitudes toward learning and the processes involved in school achievement. Unlike objective performance, PAA is inherently shaped by learners’ subjective appraisals of their abilities and learning experiences. This reasoning aligns with and extends the reciprocal effects model proposed by Huang (2011), which demonstrated that domain-specific self-concepts and objective academic achievement are mutually influential over time. Although Huang focused on objective performance, it is reasonable to hypothesize that that self-beliefs play an equally or even more direct role in shaping students’ subjective perception of that performance (i.e., PAA), given that PAA is inherently filtered through self-appraisal. In the present study, PAA is operationalized as students’ self-perceived EAP-related competence across four core skills—listening, speaking, reading, and writing—serving as a context-sensitive proxy for their overall academic achievement.

Building on this domain-specific conceptualization, EAP-related PAA is adopted as the primary outcome variable for three key reasons. First, in EMI settings where learning is mediated through language, students’ perceptions of their ability to perform EAP-related tasks (e.g., understanding lectures or engaging in discussions) offer a direct reflection of their academic functioning, avoiding inconsistencies associated with institutional grading practices. Second, from the perspective of Social Cognitive Theory (SCT), self-evaluations of competence are central to motivation and learning behavior, making EAP-related PAA theoretically aligned with the mechanisms under investigation. Third, prior research has consistently demonstrated that academic self-concept—a core component of PAA—is positively correlated with academic achievement and predicts subsequent academic outcomes (Valentine et al., 2004; Guay et al., 2003; Marsh and Craven, 2006; Jaiswal and Choudhuri, 2017). Together, these considerations support the use of EAP-related PAA as a valid and meaningful indicator of academic adaptation in EMI context.

The present study draws on SCT to explain how environmental and individual factors jointly shape students’ perceptions of EAP-related PAA. From an environmental perspective, teacher support has been shown to be a critical contextual factor that promotes students’ academic motivation and engagement (Skinner and Belmont, 1993). From an individual perspective, self-efficacy, defined as individuals’ beliefs in their capability to successfully perform academic tasks, plays a central role in determining persistence, effort, and resilience in learning (Bandura, 1977). Furthermore, academic engagement has been identified as an important behavioral mechanism linking external supports and students’ academic outcomes (Reeve, 2012). Within the SCT framework, these environmental, personal, and behavioral factors interact to shape how students interpret and evaluate their academic experiences.

Although extensive research has demonstrated the positive relationship between teacher support and academic performance (Ansong et al., 2024; Jiang, 2024; Ouyang and Wong, 2025), relatively little attention has been paid to how teacher support influences students’ PAA, particularly in internationalized educational contexts. Most existing studies have focused on traditional educational settings and objective indicators such as Grade Point Average (GPA), overlooking the complexities of internationalized programs such as the “4 + 0″ dual-degree model, where students must navigate both linguistic and cultural challenges within English-medium academic environments. Moreover, limited research has examined the psychological mechanisms through which teacher support influences students’ perceived academic success, particularly through key factors such as self-efficacy and academic engagement in internationalized EAP learning contexts.

Guided by SCT, the present study investigates how teacher support influences students’ EAP-related PAA in EMI context within Sino-foreign cooperative programs. Specifically, the study examines the mediating roles of self-efficacy and academic engagement in this relationship. By focusing on the learning experiences of students enrolled in “4 + 0″ dual-degree programs, this research aims to deepen understanding of the psychological mechanisms underlying academic success in cross-cultural English-medium educational environments. The findings are expected to offer insights into pedagogical practices, teacher–student interactions, and students’ academic adaptation in internationalized higher education settings.

## 2 Literature review

### 2.1 Theoretical framework

This study is grounded in SCT, which provides a comprehensive theoretical framework for understanding how environmental, personal, and behavioral factors interact to shape learning processes and outcomes. A central tenet of SCT is the principle of triadic reciprocal causation, which posits that personal, behavioral, and environmental influences operate as interlocking determinants that dynamically and bidirectionally affect one another.

Within this triadic framework, the core constructs of the present study are conceptually aligned as follows: Teacher support is conceptualized as a key environmental factor. Self-efficacy represents the personal component, referring to students’ domain-specific beliefs in their capabilities to organize and execute the courses of action required to manage academic tasks in English-medium learning environments. Academic engagement corresponds to the behavioral dimension, capturing the observable manifestation of motivation through students’ active involvement, effort, persistence, and strategic investment in learning activities. Finally, EAP-related PAA is conceptualized as a core cognitive outcome, reflecting students’ subjective evaluation and judgment of their own academic performance and competence within the specific, demanding EMI context.

### 2.2 Teacher support

PAA is a multifaceted construct shaped by the dynamic interplay of individual and contextual factors. Within this socio-educational context, teacher support has been widely recognized as a key environmental influence on students’ academic development. Conceptually, teacher support is rooted in social support theory. According to House (1981), social support can be categorized into four functional forms—emotional, instrumental, informational, and appraisal support—which represent different ways supportive relationships provide care, guidance, resources, and evaluative feedback. Building on this framework, Cohen and Wills (1985) further explained how social support buffers stress and promotes psychological well-being, which may apply to teacher support in the educational context.

In educational settings, these four dimensions have been widely adopted to conceptualize teacher support, capturing the emotional, informational, instrumental, and evaluative assistance that teachers provide to facilitate students’ learning and engagement. Empirical studies have applied and validated this multidimensional framework in measuring teacher support (e.g., Sadoughi and Hejazi, 2022). Within the broader framework of SCT (Bandura, 1986), these dimensions can be conceptualized as proximal environmental factors that are hypothesized to influence students’ PAA through their effects on learning experiences and motivational processes, while interacting with personal and behavioral determinants within a broader reciprocal framework.

Specifically, emotional support cultivates positive teacher–student relationships and a psychologically secure environment through care, respect, and trust, promoting social competence and prosocial behavior (Pakarinen et al., 2020), academic engagement and motivated learning behavior (Sadoughi and Hejazi, 2022), while its average level and consistency jointly predict academic and social outcomes (Curby et al., 2013). Instrumental support—defined as teachers’ provision of tangible assistance, such as devoting time, skills, or services to help students—is, alongside emotional support, a significant unique predictor of students’ subjective well-being (SWB) (Suldo et al., 2009). Informational support, defined as teachers’ sharing of information, guidance, or advice within a content area, serves as effective scaffolding, through which teachers can guide learners to adopt better learning strategies and thereby facilitate their learning process (Sadoughi and Hejazi, 2022). Appraisal support, defined as evaluative feedback (Malecki and Demary, 2003), refers in educational settings to teachers’ evaluative comments and guidance aimed at improving students’ academic performance. It can enable students to interpret feedback, monitor their learning process, adjust their efforts, and ultimately enhance learning outcomes (Nicol and Macfarlane‑Dick, 2006). Together, these dimensions constitute a multidimensional framework through which teacher support promotes students’ academic development and adaptation.

A growing body of research has demonstrated that teacher support plays a significant role in shaping students’ academic achievement. Meta-analytic evidence shows that teacher support is positively associated with academic performance, particularly among upper-secondary students, with emotional support often exerting stronger effects than other forms of support (Tao et al., 2022). Beyond general academic outcomes, teacher support has also been linked to achievement in specific domains. For example, it can enhance foreign language achievement by increasing students’ academic buoyancy and reducing second-language boredom (Derakhshan and Fathi, 2025), and promote mathematics achievement either directly or indirectly through students’ feedback literacy (Wu et al., 2025).

In addition to direct effects, increasing evidence indicates that teacher support influences academic achievement through various mediating mechanisms. Academic engagement has been identified as a key pathway linking teacher support to academic achievement (Chen, 2005; Zhou and Wu, 2024). Other studies highlight psychological and learning-related processes such as online learning satisfaction, academic resilience, and feedback literacy as important mediators that transmit the positive effects of teacher support on academic performance (Ali et al., 2021; Melaku et al., 2025; Wu et al., 2025). Overall, existing research suggests that teacher support contributes to academic achievement both directly and indirectly through multiple psychological and learning-related processes.

Despite compelling evidence, several research gaps remain. First, most existing studies emphasize objective academic achievement, while relatively few examine PAA, which may be more sensitive to students’ interpersonal experiences and psychological interpretations of learning success. Second, although the multidimensional nature of teacher support has been widely acknowledged, comprehensive analyses incorporating all four dimensions simultaneously remain limited. Third, empirical research on teacher support in Sino-foreign dual-degree programs, particularly within EAP education, remains scarce. Addressing these gaps can provide evidence-based guidance for designing supportive teaching practices that enhance academic adaptation and perceived success in cross-cultural higher education.

Within the SCT framework, teacher support is conceptualized as an environmental factor that influences students’ learning processes. Following the theorized directional sequence from Section 2.1, teacher support is hypothesized to shape students’ self-efficacy beliefs (personal factor), which in turn affect their academic engagement (behavioral factor) and ultimately their EAP-related PAA (outcome).

### 2.3 Self-efficacy

Self-efficacy, a central construct in SCT, refers to individuals’ subjective judgments of their capabilities to organize and execute courses of action required to attain designated performance goals (Bandura, 1986, 1997). Unlike objective indicators of skill or competence, self-efficacy reflects learners’ beliefs about what they can accomplish with their existing capabilities, functioning as a forward-looking, motivationally generative form of self-appraisal (Pajares, 1996; Schunk and Pajares, 2002). These efficacy beliefs play a critical role in regulating cognitive engagement, emotional responses, persistence, and resilience when individuals encounter academic challenges (Bandura, 1997).

In the context of foreign language learning, self-efficacy represents learners’ confidence in managing the complex cognitive, linguistic, and communicative demands associated with acquiring a new language. Empirical research has consistently demonstrated that language learners with higher self-efficacy exhibit greater persistence, reduced anxiety, and more adaptive coping strategies when facing linguistic difficulties (Mills et al., 2007; Raoofi et al., 2012). This is particularly relevant for Chinese tertiary English as a Foreign Language (EFL) students, many of whom experience a significant transition from highly structured, teacher-directed secondary education to more autonomous university learning environments. During this transition, self-efficacy functions as a vital psychological resource that enables learners to interpret academic challenges as manageable and controllable rather than threatening or indicative of fixed inability (Bandura, 1997; Pajares, 1996).

Beyond its motivational and regulatory functions, self-efficacy also plays a crucial role in shaping students’ academic self-evaluations. Attribution and appraisal-based theories posit that students’ interpretations of academic outcomes are not determined solely by objective performance. Rather, learners evaluate outcomes through cognitive appraisals, including their competence or control beliefs and causal attributions for success and failure (Weiner, 1985; Pekrun, 2006). Students with strong efficacy beliefs tend to attribute academic success to controllable factors such as effort and strategy use, thereby sustaining more positive perceptions of their competence (Bandura, 1997; Weiner, 1985). This perspective is further supported by the Reciprocal Effects Model, which posits that academic self-concept and achievement exert reciprocal influences on one another over time (Marsh and Craven, 2006). Consequently, PAA can be understood not merely as a reflection of objective performance, but as a cognitively mediated evaluative construct grounded in learners’ efficacy-related beliefs.

Importantly, self-efficacy is not a fixed trait but a context-specific and malleable belief about one’s capabilities that is shaped by social experiences and instructional influences (Bandura, 1997; Schunk and Pajares, 2002). Among these influences, teacher support represents a particularly salient source of efficacy-relevant information. Within classroom environments, teacher support, guidance, and feedback play a critical role in shaping students’ motivation and their perceptions of competence (Wentzel, 1998, 2002).

The SCT identifies social persuasion as a key source of efficacy information, suggesting that supportive feedback and encouragement from teachers can strengthen students’ beliefs in their academic capabilities (Bandura, 1997). In the specific context of Sino-foreign cooperative “4 + 0″ programs, this social persuasion functions through two distinct yet complementary mechanisms: appraisal support and emotional support. Appraisal support, manifested as constructive evaluative feedback, provides students with validated evidence of their incremental progress, thereby directly reinforcing their self-competence beliefs (Brookhart, 2008). Simultaneously, emotional support creates a psychological safety net that mitigates the academic anxiety inherently associated with intensive EMI. By reducing negative emotional arousal—another critical source of efficacy information in SCT—teachers help students maintain a more resilient self-belief system despite high-pressure academic demands. Empirical studies have consistently demonstrated that teacher support plays a crucial role in fostering students’ self-efficacy by providing emotional and instructional support as well as competence-related feedback (Huang and Wang, 2023; Liu et al., 2023). In EFL contexts specifically, recent research—including studies conducted in both traditional classrooms and emergency online settings—has further confirmed that teacher support contributes significantly to the development of language learning self-efficacy by providing both emotional security and constructive feedback that validates learners’ progress (Huang and Wang, 2023; Liu et al., 2023).

Taken together, prior research suggests that self-efficacy functions as a key psychological mechanism linking social-contextual influences and students’ academic self-evaluations. While teacher support provides important external cues and feedback that shape learners’ competence beliefs, self-efficacy represents a key internal cognitive mechanism through which these external influences are interpreted and integrated into students’ perceptions of their academic achievement (Bandura, 1997; Huang and Wang, 2023). Accordingly, self-efficacy may serve as a theoretically grounded mediating mechanism through which teacher support influences PAA, particularly within the context of Sino-foreign cooperative higher education programs, where students must continuously interpret and adapt to unfamiliar academic expectations.

From the perspective of SCT, self-efficacy represents a core personal factor that, consistent with the theorized directional sequence from Section 2.1, mediates the influence of environmental support (teacher support) on behavioral engagement and subsequent academic outcomes.

### 2.4 Academic engagement

Academic engagement is widely recognized as a central construct for understanding students’ learning processes and academic outcomes. Originating from the broader literature on school engagement, it reflects students’ investment of effort, attention, and commitment in learning activities (Newmann, 1992). Building on this perspective, engagement is commonly conceptualized as a multidimensional construct comprising behavioral, emotional, and cognitive components (Fredricks et al., 2004). Behavioral engagement refers to students’ participation and effort in academic tasks, emotional engagement captures their affective responses to learning and classroom interactions, and cognitive engagement involves the use of deep learning strategies and self-regulation to master complex content. More recently, the notion of agentic engagement has been introduced, highlighting students’ proactive contributions to their learning, such as asking questions and expressing preferences (Reeve and Tseng, 2011; Reeve, 2013).

Academic engagement is increasingly understood as context-dependent, shaped by the interaction between student characteristics and institutional factors, and is central to explaining differences in learning experiences and academic success (Kahu, 2013; Kahu and Nelson, 2018). In higher education, and particularly in EFL contexts, academic engagement is critical because learners face challenges such as linguistic complexity, unfamiliar academic discourse, and high-stakes assessments. Research in second language learning has conceptualized engagement as multidimensional, encompassing cognitive, behavioral, emotional, and social aspects (Philp and Duchesne, 2016). From a process-oriented perspective, engagement links motivational dynamics to learning behaviors and outcomes (Noels et al., 2020). In this regard, self-efficacy may be understood as a “cognitive gateway” to sustained learning efforts. Drawing on SCT, students with stronger beliefs in their competence are more likely to invest strategic effort and demonstrate resilience when encountering academic challenges (Bandura, 1997). Empirical research further suggests that self-efficacy is a significant predictor of academic engagement, facilitating students’ active involvement in learning processes (Ouweneel et al., 2013).

Beyond persistence, high self-efficacy also fosters agentic engagement, characterized by students’ proactive contribution to the instructional process, such as seeking clarification, expressing preferences, and offering input rather than remaining passive recipients of knowledge (Reeve and Tseng, 2011; Reeve, 2013). This proactive form of engagement has been shown to be closely associated with motivational resources, including self-efficacy, and contributes uniquely to academic achievement (Reeve, 2013; Lei et al., 2018). Empirical studies further indicate that deeper forms of engagement can support learners’ self-regulated learning and persistence in academic language tasks (Abdollahzadeh et al., 2022).

A growing body of evidence suggests that academic engagement is positively associated with students’ perceived learning and academic achievement. Cognitive and emotional engagement appear to strengthen perceived learning, whereas behavioral engagement is more strongly linked to academic performance (Navarro et al., 2024; Wong et al., 2024), and higher engagement levels correspond to more positive evaluations of learning effectiveness (Panigrahi et al., 2021).

Teacher support is a key antecedent of academic engagement. Drawing on the self-system model of motivational development, Skinner et al. (2008) showed that involvement, structure, and autonomy support enhance students’ perceptions of competence, autonomy, and relatedness, which in turn promote engagement and reduce disaffection. In EFL settings, empirical studies confirm that academic and emotional support from teachers can directly or indirectly foster engagement through motivational and emotional pathways (Liu et al., 2023; Sadoughi and Hejazi, 2021). Considering that engagement is positively linked to perceived achievement, it may act as a mediator in the relationship between teacher support and students’ academic outcomes.

Building on this evidence, the present study situates academic engagement within a Sino-foreign cooperative “4 + 0” dual-degree program with EMI and internationally aligned curricula. In this cross-cultural context, teacher support, particularly guidance on assessment interpretation, metacognitive strategies, and cross-cultural adaptation, may play a critical role in promoting academic engagement. Accordingly, this study examines whether academic engagement mediates the effect of teacher support on students’ EAP-related PAA, thereby extending research on academic engagement to EMI-based cooperative higher education.

Within the SCT framework, academic engagement is viewed as the behavioral dimension that, in line with the theorized directional sequence from Section 2.1, links personal beliefs (self-efficacy) to outcome perceptions (EAP-related PAA).

### 2.5 The present study

Drawing on SCT, particularly its core principle of triadic reciprocal causation, the present study explores the mechanisms through which teacher support influences students’ EAP-related PAA within the “4 + 0″ dual-degree model—a prevalent Sino-foreign cooperative program configuration—in EMI context.

Within this framework, teacher support constitutes a critical environmental resource. Scaffolding (Wood et al., 1976) and formative feedback (Nicol and Macfarlane-Dick, 2006) provide the necessary structured guidance that helps students clarify assessment expectations and reduce ambiguity regarding performance standards. Perceived pedagogical care and teacher responsiveness foster a supportive relational climate that enhances students’ academic security and their prosocial and academic motivation (Wentzel, 1997). Consistent with SCT, such supportive instructional behaviors cultivate students’ self-efficacy by providing proximal mastery experiences, positive social persuasion, and enhanced affective regulation. As students gain greater clarity and confidence in managing complex academic demands, their efficacy beliefs are progressively strengthened.

Self-efficacy functions as a pivotal cognitive gateway, translating perceived environmental support into sustained academic engagement. Students who perceive themselves as capable of handling academic challenges are more likely to invest effort strategically, persist when encountering difficulties, and engage in self-regulated learning behaviors (Schunk and Pajares, 2009). In foreign language learning contexts, efficacy beliefs influence how students interpret academic challenges and attribute performance outcomes (Hsieh and Schallert, 2008), while engagement reflects the observable behavioral manifestation of these motivational processes. Empirical evidence indicates that supportive teacher behaviors, such as involvement and structure, are positively associated with students’ engagement over time (Skinner and Belmont, 1993). Student engagement is commonly conceptualized as comprising behavioral, emotional, and cognitive dimensions (Fredricks et al., 2004).

Through sustained engagement, students accumulate mastery experiences that provide important information for evaluating their academic progress. In this framework, academic engagement can be regarded as the behavioral manifestation of academic success, as continuous behavioral and cognitive involvement enables learners to generate concrete evidence of their own learning development. These accumulated mastery experiences—considered the most influential source of efficacy beliefs (Bandura, 1997)—also serve as an important basis for students’ evaluative judgments of their academic standing. As students witness their own tangible progress through active participation, these experiences provide an objective reference point for self-appraisal, ultimately enhancing their PAA.

Recent empirical studies provide support for such sequential mediation mechanisms. For example, teacher support has been shown to enhance learning engagement through the intermediary role of self-efficacy (Liang et al., 2026). Similarly, supportive instructional environments can foster students’ academic engagement by strengthening their efficacy beliefs, suggesting a pathway through which environmental support is translated into sustained behavioral involvement (Huang and Wang, 2023; Skinner et al., 2008). These findings provide convergent evidence for a theoretically ordered mediation process linking environmental resources, motivational beliefs, and engagement in higher education.

Furthermore, the present study also posits a direct link between teacher support and EAP-related PAA. This assumption is grounded in scaffolding theory, which emphasizes the role of instructional guidance in structuring learners’ understanding of task demands (Wood et al., 1976). Beyond its indirect influence through motivational and behavioral processes, teachers’ instrumental and informational support—such as clarifying assessment criteria and providing task-specific guidance—may exert a “clarification effect” by reducing task ambiguity. In highly structured academic contexts, this reduction of uncertainty enables students to form more accurate and often more positive evaluations of their own performance. As a result, teacher support may directly enhance students’ EAP-related PAA, independent of their underlying motivational states.

**Figure 1:**
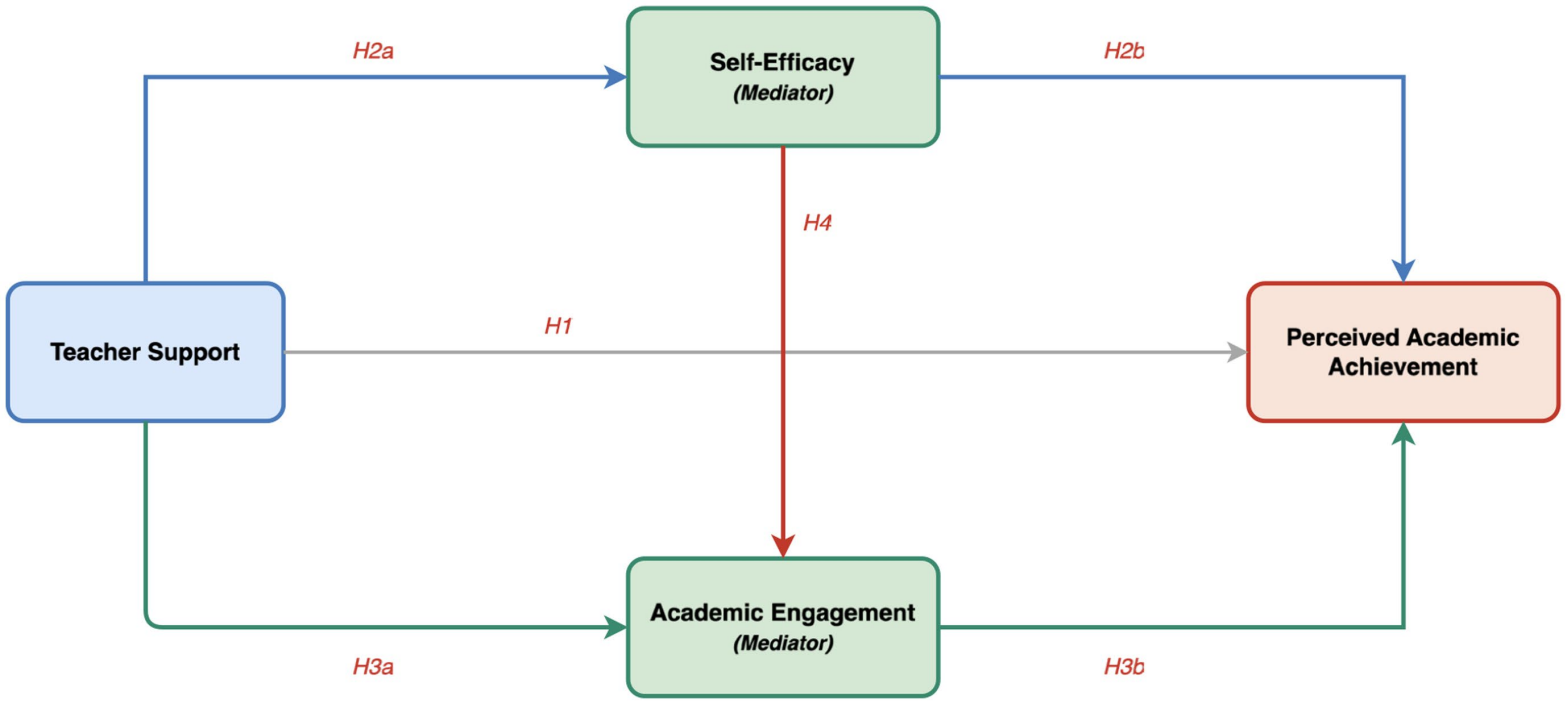
Theoretical model.

Based on this framework, the following hypotheses are proposed (see Figure 1):

*H1*: Teacher support positively correlates with students’ EAP-related PAA in EMI context.

*H2*: Students’ self-efficacy mediates the relationship between teacher support and EAP-related PAA in EMI context.

*H3*: Students’ academic engagement mediates the relationship between teacher support and EAP-related PAA in EMI context.

*H4*: Students’ self-efficacy and academic engagement sequentially mediate the relationship between teacher support and EAP-related PAA in EMI context.

## 3 Methodology

### 3.1 Instruments

The survey utilized in this study consisted of five sections: (1) demographic information, (2) teacher support, (3) self-efficacy, (4) academic engagement, and (5) EAP-related PAA. All multi-item scales, except where noted, were measured on a seven-point Likert scale ranging from 1 (*strongly disagree*) to 7 (*strongly agree*). The original English versions of the scales were translated into Chinese. The translation process involved forward and back translations performed independently by two professional translators, after which the versions were compared and refined. To ensure linguistic and cultural appropriateness for the target population, minor adaptations were made to the wording of some items. To identify and exclude responses from inattentive participants, two instructed-response items (e.g., “Please select 3 for this item to show that you are reading carefully” and “I understand this question, please select 5 for this item”) were embedded in the self-efficacy and perceived academic achievement sections, respectively.

Teacher support was measured using the Perceived Teacher Support Scale (PTSS) developed by Wu et al. (2024). This 25-item scale assesses students’ perceptions of support from their teachers across four dimensions: instrumental support (7 items), emotional support (6 items), informational support (7 items), and appraisal support (5 items). A sample item is “My teachers will take time to help me when I need help for my study” (Wu et al., 2024).

Self-Efficacy was assessed using an adapted version of the Questionnaire of English Self-Efficacy (QESE), originally developed by Wang et al. (2014). The original 32-item scale measures English self-efficacy across four skills: listening (8 items), speaking (8 items), reading (8 items), and writing (8 items). For the purpose of this study, the original interrogative sentences were rephrased into declarative statements to maintain consistency with the rest of the survey instrument. An example of an adapted item is “I can understand stories told in English,” which corresponds to an item in the original listening self-efficacy subscale.

Academic Engagement was measured using the scale developed by Reeve and Tseng (2011), which conceptualizes student engagement as a four-dimensional construct. The scale consists of 22 items assessing four aspects of engagement: behavioral engagement (5 items), agentic engagement (5 items), cognitive engagement (8 items), and emotional engagement (4 items). Behavioral engagement refers to students’ attention and effort in learning activities (e.g., “I listen carefully in class”). Agentic engagement captures students’ constructive contribution to the flow of instruction (e.g., “During class, I express my preferences and opinions”). Cognitive engagement involves the use of sophisticated learning strategies and metacognitive self-regulation (e.g., “When doing schoolwork, I try to relate what I’m learning to what I already know”). Emotional engagement reflects students’ affective involvement during class (e.g., “When I am in class, I feel curious about what we are learning”).

Given that academic learning in EMI settings is inherently language-mediated, students’ ability to comprehend, communicate, and produce academic content in English constitutes a core dimension of academic performance. Accordingly, these self-perceived competencies serve as a theoretically grounded and context-sensitive proxy for PAA. On this basis, EAP-related PAA was operationalized using the Self-Perceptions of Language Use scale developed by Uchihara and Harada (2018). This 29-item instrument is designed to gauge students’ self-perceived performance in EMI context across four language skills: listening (6 items), speaking (10 items), reading (8 items), and writing (5 items). A sample item from the listening subscale is “I can understand the content of discussion” (Uchihara and Harada, 2018, p. 586).

### 3.2 Data collection

To ensure adequate sample size, an *a priori* power analysis was performed with G*Power (Faul et al., 2007). Based on a multiple regression model with three predictors, the analysis showed that a minimum sample of 77 participants was required to detect a medium effect size (*f*^2^ = 0.15) with 80% power at a significance level of *α* = 0.05 (Memon et al., 2020).

Data were collected between December 2025 and January 2026 using an online survey administered through Wenjuanxing^1^, a widely used questionnaire platform in China. Based on a convenience sampling method, this study recruited 670 students from the “4 + 0” academic programs at a comprehensive university in China. This sampling approach was employed due to its practicality and efficiency in accessing the target population within the given research constraints. It is important to note that the “4 + 0” programs at this specific university are exclusively offered in three engineering disciplines. According to statistics from Ministry of Education of China (2025), engineering disciplines (such as mechanical design and manufacturing and its automation, electrical engineering and its automation, civil engineering) have long been the leading ones in terms of the number of cooperative education programs, and majors like computer science and technology, accounting, and electronic information engineering also rank among the popular cooperative education majors. However, this university’s “4 + 0” programs only cover a subset of these top-ranked professional fields (limited to three engineering disciplines), so the sample may exhibit certain characteristics that limit its representativeness of the broader population of students in similar internationalized programs. After briefly explaining the research purpose and obtaining written informed consent, the instructors provided participants with a link to the online survey. Students who agreed to participate voluntarily completed the questionnaire.

Of the collected responses, 170 were excluded from the analysis due to invalid response patterns, such as excessively short completion time (more than three standard deviations below the mean) or uniform answers across all items. Consequently, 500 valid questionnaires were retained for further analysis, yielding a valid response rate of 74.6%.

### 3.3 Data analysis

Data analysis was conducted using SPSS 27 and AMOS 26. According to a widely adopted two-step procedure (Hair et al., 2010), visual data inspection and structural equation modeling in AMOS are generally performed in the following sequence. First, the reliability and validity of each latent construct should be evaluated by examining the measurement model. This initial stage, which includes assessments of reliability and validity, provides the necessary foundation for subsequent analyses. Second, after the criteria for reliability and validity are met, the overall model fit is examined to ensure that the estimated paths reflect meaningful relationships. Subsequently, a detailed analysis of the relationships among constructs is carried out, including systematic validation and mediation analysis within the structural model (Hair et al., 2012).

## 4 Results

### 4.1 Common method bias

As with any study employing self-reported measures, the potential for common method bias (CMB) exists due to factors such as consistency motif and social desirability (Podsakoff et al., 2003; Podsakoff and Organ, 1986). Given that CMB may threaten the validity of research findings, this study implemented both procedural and statistical remedies to control and assess its influence. Procedurally, the survey was designed following the principle of respondent anonymity. Several strategies were adopted to mitigate CMB, including counterbalancing item sequence, randomizing item order, and refining scale items to reduce ambiguity.

For statistical control, two complementary statistical approaches were employed. First, Harman’s single-factor test was performed on all measurement items without rotation. The results indicated that the first factor accounted for 32.6% of the total variance, which is below the commonly recommended threshold of 40% (Tang and Wen, 2020), providing an initial indication that common method bias was not a predominant concern. Second, and more rigorously, an unmeasured latent method factor (ULMF) was incorporated into the structural equation model following established procedural recommendations (Podsakoff et al., 2003) to statistically disentangle method variance from substantive construct relationships. The model comparison revealed that the inclusion of the method factor did not yield a substantial improvement in model fit (ΔGFI = 0.017, ΔCFI = 0.014, ΔTLI = 0.010, ΔRMSEA = 0.007, ΔSRMR = 0.013), and the substantive factor loadings remained significant. These findings collectively suggest that common method bias is unlikely to have been a primary driver of the observed relationships in this study (Richardson et al., 2009).

### 4.2 Demographic analysis

As presented in Table 1, the sample exhibits two notable characteristics: a high proportion of male participants (71.8%) and an exclusive background in engineering disciplines. This composition is reflective of the gender distribution and program offerings within the specific “4 + 0” dual-degree programs at the target university, which are common in many Chinese cooperative education models. However, it is important to acknowledge that this profile may introduce sample bias. The predominance of males and the absence of students from non-engineering fields mean that the experiences, perceptions, and psychological mechanisms captured in this study may not be fully representative of the broader, more diverse population of students in Sino-foreign cooperative programs, particularly those with more balanced gender ratios or in different academic domains.

**Table 1:** Demographic features of participants.

Demographics	Classification	Number	Percent
Gender	Female	141	28.2
	Male	359	71.8
Level of Study	Freshman	316	63.2
	Sophomore	184	36.8
Discipline	Computer Science and Technology	175	35.0
	Electronic Information Engineering	154	30.8
	Communication Engineering	171	34.2

### 4.3 Validity and reliability

Relying exclusively on Cronbach’s alpha to establish validity in a SEM falls short of providing a comprehensive evaluation. To assess the construct validity, two key indicators were carefully considered: CR and the average variance extracted (AVE). In SEM, the AVE and CR benchmarks are 0.50 (Fornell and Larcker, 1981) and 0.70 (Hair et al., 2011), respectively. As shown in Table 2, all CR values for the constructs exceeded 0.88, the AVE values exceeded 0.59, affirming a satisfactory level of internal consistency and acceptable reliability across the measured constructs.

**Table 2:** Results of the reliability and convergent validity of the measurement model.

Dimension	CR	AVE	Cronbach’s alpha
IS	0.911	0.596	0.909
ES	0.930	0.690	0.929
IfS	0.917	0.614	0.917
AS	0.894	0.629	0.892
SEL	0.922	0.598	0.917
SES	0.947	0.693	0.946
SER	0.942	0.669	0.941
SEW	0.947	0.691	0.946
BE	0.886	0.609	0.881
AgE	0.929	0.725	0.928
CE	0.952	0.713	0.952
EE	0.923	0.752	0.921
LS	0.931	0.693	0.929
SS	0.951	0.660	0.951
RS	0.948	0.694	0.947
WS	0.947	0.783	0.947
TS	0.895	0.682	0.865
SE	0.885	0.660	0.868
AE	0.919	0.741	0.891
PAA	0.935	0.784	0.919

TS, teacher support; IS, instrument support; ES, emotional support; IfS, informational support; AS, appraisal support; SE, self-efficacy; SEL, self-efficacy for listening; SES, self-efficacy for speaking; SER, self-efficacy for reading; SEW, self-efficacy for writing; AE, academic engagement; BE, behavioral engagement; AgE, agentic engagement; CE, cognitive engagement; EE, emotional engagement; PAA, perceived academic achievement; LS, listening skill; SS, speaking skill; RS, reading skill; WS, writing skill.

Validity testing primarily examines the discriminant validity among variables, which refers to the low correlation and significant differences between latent variables. This can be evaluated by comparing the square root of the AVE with the correlation coefficients between the variables. Following Fornell and Larcker (1981), if the correlation coefficient between a variable and another is smaller than the square root of the AVE for that variable, then it has good discriminant validity. In Table 3, the bold values on the diagonal represent the square root of the AVE for each construct, while the off-diagonal values represent the inter-construct correlation coefficients. The square root of the AVE for each construct is greater than its correlations with all other constructs. This pattern indicates that the measurement model possesses appropriate discriminant validity.

**Table 3:** Discriminant validity.

Construct	CR	AVE	MSV	MaxR(H)	TS	SE	AE	PAA
TS	0.867	0.620	0.200	0.872	**0.787**			
SE	0.872	0.630	0.382	0.881	0.447***	**0.794**		
AE	0.898	0.687	0.353	0.902	0.445***	0.595***	**0.829**	
PAA	0.920	0.741	0.382	0.923	0.383***	0.618***	0.539***	**0.861**

CR, composite reliability; AVE, average variance extracted; MSV, max shared variance; MaxR(H), maximal reliability; Square roots of AVE are displayed in bold on the diagonal; Off-diagonal values are the Pearson correlations between the constructs. ***p < 0.001.

### 4.4 SEM fit indices

The model fit was assessed using multiple fit indices based on established benchmarks (Bagozzi and Yi, 1988; Hair et al., 2017; Hayduk, 1987; Hu and Bentler, 1998). In line with recommendations by Kline (2016, p. 269), a set of key fit statistics is reported, including the chi-square test, degrees of freedom, *p*-value, the Root Mean Square Error of Approximation (RMSEA) with its 90% confidence interval, the Comparative Fit Index (CFI), and the Standardized Root Mean Square Residual (SRMR).

As shown in Table 4, the model results are as follows: *χ*^2^ = 322.407, df = 98, *p* < 0.001; RMSEA = 0.068 (90% CI, 0.060–0.076), SRMR = 0.048. Moreover, the approximate fit indices all reached or exceeded the commonly accepted threshold of 0.90: CFI = 0.957, NFI = 0.940, RFI = 0.927, IFI = 0.958, TLI = 0.948, GFI = 0.925. Together, these indices support the appropriateness of the model in representing the collected data and validating the hypothesized relationships among the variables.

**Table 4:** Model fit indicators.

Fit indices	Values
Chi-square	322.407
df	98
*p*	<0.001
RMSEA (90% CI)	0.068 (0.060–0.076)
CFI	0.957
SRMR	0.048
NFI	0.940
RFI	0.927
IFI	0.958
TLI	0.948
GFI	0.925

### 4.5 Mediation analysis

**Figure 2:**
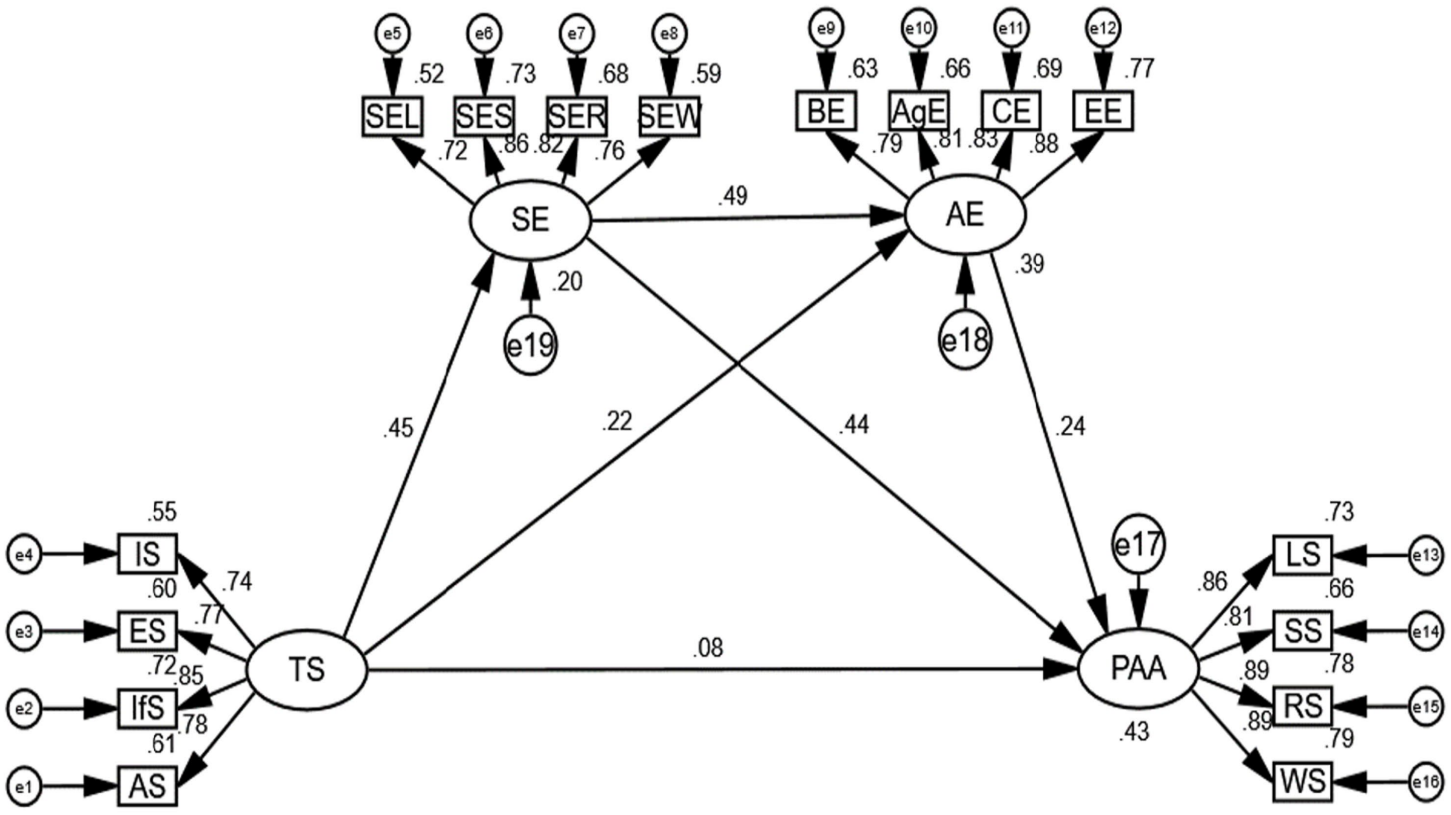
Structural model diagram.

To investigate the indirect effects of the dependent variable (PAA) through the mediators (SE, AE), we performed percentile bootstrapping and bias-corrected percentile bootstrapping at a 95% confidence interval with 5,000 bootstrap samples (see Figure 2). Following the suggestions of Preacher and Hayes (2008), we calculated the lower and upper bounds of the confidence interval to test the significance of the indirect effects.

As shown in Table 5, the bootstrap test results confirmed the existence of a positive and significant mediating effect for SE and AE (the combined mediation of SE and AE) between TS and PAA (point estimate = 0.417, BC 95% CI [0.315, 0.549], Percentile 95% CI [0.303, 0.532]). Additionally, there were positive and significant mediating effects for SE between TS and PAA (point estimate = 0.269, BC 95% CI [0.185, 0.376], Percentile 95% CI [0.176, 0.367]) and for AE between TS and PAA (point estimate = 0.075, BC 95% CI [0.033, 0.140], Percentile 95% CI [0.027, 0.129]). H2 and H3 are supported. There was also a significant chain-mediation effect of SE and AE between TS and PAA (TS → SE → AE → PAA) with a point estimate of 0.074 (BC 95% CI [0.039, 0.124], Percentile 95% CI [0.036, 0.115]). Therefore, H4 is supported.

**Table 5:** Mediation analysis results.

Relations	Point estimation	Product of coefficients		Bootstrapping			
				BC 95% CI		Percentile 95% CI	
		SE	*Z*	Lower	Upper	Lower	Upper
Indirect effects							
1. TS → SE → AE → PAA	0.074	0.020	3.700	0.039	0.124	0.036	0.115
2. TS → SE → PAA	0.269	0.049	5.490	0.185	0.376	0.176	0.367
3. TS → AE → PAA	0.075	0.026	2.885	0.033	0.140	0.027	0.129
Total indirect	0.417	0.059	7.068	0.315	0.549	0.303	0.532
Direct paths							
TS → PAA	0.108	0.076	1.421	−0.031	0.270	−0.027	0.274
TS → SE	0.486	0.052	9.346	0.392	0.604	0.386	0.592
TS → AE	0.212	0.052	4.077	0.115	0.324	0.110	0.317
SE → AE	0.431	0.057	7.561	0.328	0.554	0.329	0.555
SE → PAA	0.553	0.082	6.744	0.400	0.727	0.393	0.714
AE → PAA	0.353	0.084	4.202	0.184	0.513	0.170	0.508
Contrasts							
1–2	−0.195	0.052	−3.750	−0.309	−0.100	−0.305	−0.097
1–3	−0.001	0.026	−0.038	−0.056	0.049	−0.053	0.052
2–3	0.194	0.059	3.288	0.089	0.325	0.084	0.320

In terms of direct effects, the direct path from TS to PAA had a point estimate of 0.108 (SE = 0.076, *Z* = 1.421). The bias-corrected (BC) 95% confidence interval was [−0.031, 0.270], and the percentile 95% confidence interval was [−0.027, 0.274]. Since the confidence intervals included zero, the direct effect of TS on PAA was not statistically significant after accounting for the mediators. These results suggest that the effect of TS on PAA is mainly transmitted through the mediation pathways, and the model is a complete mediation model, as the direct effect is non-significant and the majority of the total effect is explained by the indirect (mediation) paths.

Regarding the pairwise comparisons of the indirect effects, the contrast analysis indicated the following: The difference between the chain-mediation effect (SE → AE) and the simple mediation effect via SE alone was negative and statistically significant (point estimate = −0.195, *Z* = −3.750, BC 95% CI [−0.309, −0.100], Percentile 95% CI [−0.305, −0.097]), which suggests that the indirect effect through SE alone was significantly greater than the indirect effect transmitted via the sequential pathway of SE and AE. The difference between the chain-mediation effect (SE → AE) and the simple mediation effect through AE alone was not statistically significant (point estimate = −0.001, *Z* = −0.038, BC 95% CI [−0.056, 0.049], Percentile 95% CI [−0.053, 0.052]), as both bias-corrected and percentile confidence intervals contained zero. The difference between the two simple mediation effects, SE alone versus AE alone, was positive and significant (point estimate = 0.194, *Z* = 3.288, BC 95% CI [0.089, 0.325], Percentile 95% CI [0.084, 0.320]), indicating that the indirect effect via SE was significantly stronger than that via AE in linking teacher support to PAA.

## 5 Discussion

### 5.1 Teacher support and EAP-related PAA

For students in engineering-focused cohorts within Sino-foreign cooperative “4 + 0” dual-degree programs, the present study examined the relationship between teacher support and students’ EAP-related PAA. The findings reveal a significant positive association between teacher support and EAP-related PAA, supporting H1. This result is consistent with previous research demonstrating that supportive teacher–student relationships play a critical role in promoting students’ academic development and learning outcomes (Ansong et al., 2024; Tao et al., 2022). While much of the existing literature has focused primarily on objective indicators such as grade point average or standardized test scores, the present study extends this line of inquiry by highlighting the importance of teacher support in relation to students’ EAP-related PAA in EMI context.

From the perspective of SCT, environmental factors such as teacher support are theorized to interact with personal beliefs and behavioral processes in relation to learning outcomes (Bandura, 1986). In classroom contexts, supportive instructional practices provide important informational and emotional cues that help students interpret academic demands and evaluate their learning progress. When teachers offer encouragement, constructive feedback, and practical assistance, students are more likely to perceive learning tasks as manageable and attainable. Such supportive interactions can reduce uncertainty regarding academic expectations and foster a sense of competence, which may ultimately contribute to more positive self-perceptions of their academic performance in EMI context.

This relationship may be particularly salient in internationalized EAP learning environments. Students enrolled in “4 + 0” programs often encounter unfamiliar academic discourse conventions, EMI, and demanding assessment systems aligned with international standards (Macaro et al., 2018). Under these circumstances, EAP instructors frequently function not only as language teachers but also as academic mediators who help students interpret disciplinary expectations and adapt to cross-cultural learning environments. The present findings are consistent with the view that students who perceive stronger teacher support in these contexts, also tend to report a positive appraisal of their ability to perform academic tasks through English. In this sense, teacher support plays a crucial role in facilitating students’ psychological adaptation to internationalized higher education settings.

### 5.2 The mediating role of self-efficacy

The results further demonstrate that self-efficacy significantly mediates the relationship between teacher support and EAP-related PAA, supporting H2. Specifically, students who perceived higher levels of teacher support reported stronger beliefs in their academic capabilities, which were, in turn, associated with higher levels of EAP-related PAA in EMI context. This finding aligns with a substantial body of literature indicating that self-efficacy serves as a key psychological mechanism through which contextual influences affect students’ academic outcomes (Huang and Wang, 2023; Liu et al., 2023).

According to SCT, self-efficacy beliefs develop through several sources, including mastery experiences, social persuasion, and affective states (Bandura, 1997). Teacher support contributes to these sources in multiple ways. For instance, informational and instrumental support could facilitate students’ successful completion of challenging academic tasks, which may provide mastery experiences conducive to stronger efficacy beliefs. Emotional support and positive feedback serve as forms of social persuasion that reinforce students’ confidence in their academic abilities. In addition, supportive classroom environments can reduce anxiety and foster positive emotional experiences, which further enhance students’ perceptions of competence.

In EAP learning contexts, efficacy beliefs are particularly important because students must simultaneously process complex disciplinary content and communicate in a second language. These dual demands often create uncertainty and cognitive pressure. When teacher support helps students clarify academic expectations and provides guidance for completing tasks in English, learners may feel more capable of managing these challenges. Consequently, students with stronger efficacy beliefs in this context also reported interpreting their academic experiences more positively and evaluating their performance with greater confidence.

Interestingly, the mediation analysis revealed that the indirect effect through self-efficacy was stronger than the mediation pathway through academic engagement alone. This finding highlights the importance of students’ cognitive interpretations of their capabilities in shaping EAP-related PAA. In other words, before students become behaviorally engaged in learning activities, their beliefs about whether they are capable of succeeding may already influence how they evaluate their performance in EMI-based academic tasks.

### 5.3 The mediating role of academic engagement

In addition to self-efficacy, the results indicate that academic engagement also mediates the relationship between teacher support and EAP-related PAA, supporting H3. Students who perceived higher levels of teacher support reported greater engagement in learning activities, which was further associated with stronger perceptions of their academic performance in EMI context. This finding aligns with prior research identifying academic engagement as a pivotal mediator that links supportive learning environments to academic outcomes (Fredricks et al., 2004; Skinner et al., 2008).

Teacher support may be linked to academic engagement through several theoretical mechanisms. Emotional support can foster a sense of belonging and psychological safety in the classroom, encouraging students to participate more actively in learning activities. Informational support clarifies task requirements and provides guidance on how to approach complex academic tasks, enabling students to invest their effort more effectively. Instrumental support offers practical assistance and learning resources, while appraisal support helps students monitor their progress through feedback and evaluation. Together, these forms of support create a classroom environment that encourages sustained participation and cognitive investment in academic tasks.

Within EAP and EMI contexts, engagement is particularly crucial because language learning requires continuous practice, interaction, and strategic effort. Students who actively participate in classroom discussions, employ cognitive learning strategies, and contribute constructively to the flow of instruction are more likely to perceive themselves as making meaningful academic progress. Behavioral and cognitive investment in learning tasks provides tangible evidence of learning advancement, which may strengthen students’ self-perceived competence in academic language use.

Thus, from a theoretical standpoint, academic engagement can be viewed as a behavioral manifestation of motivational processes that are part of the observed association between teacher support and students’ EAP-related PAA in EMI context.

### 5.4 Sequential mediation of self-efficacy and academic engagement

Beyond the independent mediation effects, the study also identified a significant sequential mediation pattern linking teacher support, self-efficacy, academic engagement, and EAP-related PAA. This finding supports H4 and provides empirical evidence for the theoretically ordered process proposed by SCT, which emphasizes the reciprocal interaction among environmental influences, personal beliefs, and behavioral processes (Bandura, 1986).

This sequential pattern is interpretable within the theoretical framework of SCT. According to the theory, teacher support could influence students’ internal motivational beliefs. For example, teacher encouragement and feedback might relate to students’ development of confidence. Such confidence, in turn, could be linked to increased effort and persistence in learning, reflected as higher engagement. Through sustained engagement, students accumulate mastery experiences and develop deeper involvement in learning tasks, which subsequently contribute to more positive self-evaluations of their academic performance in EMI context.

Consistent with the SCT-based theorized directional sequence from Section 2.1, our findings indicate that self-efficacy (personal factor) mediates the relationship between teacher support (environmental factor) and academic engagement (behavioral factor), which in turn relates to EAP-related PAA (outcome). Self-efficacy represents a central form of perceived control over academic tasks. When students believe they have the capability to succeed, they are more likely to approach learning activities with persistence and enthusiasm, thereby increasing their engagement in academic tasks.

The contrast analysis further revealed that the mediation effect through self-efficacy alone was stronger than the pathway through engagement alone. This pattern suggests that motivational beliefs may serve as a more fundamental determinant of EAP-related PAA than behavioral engagement alone. Nevertheless, the significant sequential mediation pathway indicates that engagement remains an essential behavioral channel through which efficacy beliefs translate into students’ perceptions of learning success.

Overall, for students in engineering-focused cohorts within Sino-foreign cooperative programs, these findings contribute to a more nuanced understanding of the relationships between teacher support and students’ academic experiences in internationalized higher education contexts. By identifying both independent and sequential mediation patterns, the study illustrates a model in which perceptions of environmental support are associated with motivational beliefs and behavioral engagement, which in turn are linked to students’ perceptions of their performance in EMI-based academic activities.

## 6 Implications and limitations

### 6.1 Implications

For students in engineering-focused cohorts within Sino-foreign “4 + 0” programs, the findings of this study offer several practical implications for instructional practices in internationalized higher education, particularly in similar programs adopting EMI.

First, the results highlight the importance of providing multidimensional teacher support in EAP classrooms. Given the observed direct and indirect associations between teacher support and students’ EAP-related PAA, instructors are encouraged to integrate emotional, informational, instrumental, and appraisal support in their teaching practices. Creating a supportive classroom climate characterized by empathy, respect, and responsiveness could contribute to a stronger sense of belonging and psychological security among students.

Second, the results underscore the potential value of attending to students’ self-efficacy in academic language learning. In the context of this study, self-efficacy was identified as a key mediating factor. Therefore, instructional practices that aim to cultivate students’ confidence in managing academic tasks in English appear warranted. Teachers might support the development of efficacy beliefs by, for example, providing scaffolded tasks, offering constructive formative feedback, highlighting incremental progress, and encouraging students to attribute outcomes to effort and effective strategies.

Third, attention to fostering students’ academic engagement in EAP learning environments is suggested by the findings. The results indicate that engagement is linked to EAP-related PAA in EMI context, and it may be supported through pedagogical approaches such as interactive classroom activities, opportunities for discussion and collaboration, and strategies that encourage students to express their ideas. Clear explanations of assessment criteria and academic expectations may also help students participate more actively and strategically in learning tasks.

Finally, the findings suggest broader implications for professional development and curriculum design in similar internationalized higher education. For institutions offering “4 + 0” programs, it may be beneficial to consider training programs that help instructors understand the relationships observed in this study, namely how perceptions of teacher support are associated with students’ motivation, engagement, and perceptions of academic performance. Integrating supportive pedagogical practices into EAP instruction could be a promising approach to supporting students’ academic adaptation and fostering more positive learning experiences in cross-cultural academic environments.

### 6.2 Limitations

Despite its contributions, this study has several limitations that should be acknowledged.

First, the study adopted a cross-sectional research design, which limits the ability to draw causal conclusions regarding the relationships among teacher support, self-efficacy, academic engagement, and EAP-related PAA in EMI context. Future studies could employ longitudinal or cross-lagged designs to examine how these variables interact and develop over time.

Second, the data were collected using self-reported questionnaires, which may introduce potential biases such as social desirability or common method variance. Although procedural and statistical methods were implemented to mitigate these issues, future research could incorporate multiple data sources, including teacher assessments, classroom observations, or objective academic performance indicators.

Third, the sample was drawn from a single comprehensive university in China, which may limit the generalizability of the findings to other institutional contexts. Internationalized higher education programs vary widely in terms of curriculum design, language policies, and student demographics. Future research could examine similar models across multiple institutions or countries to test the robustness of the proposed relationships.

Fourth, the present study did not statistically control for key demographic variables, such as gender, year of study, and academic discipline, in the hypothesized model. While the sample demographics are reported, their potential confounding effects on the relationships among teacher support, self-efficacy, academic engagement, and EAP-related PAA were not accounted for. Future research could strengthen the robustness of the findings by incorporating these demographic factors as control variables in the structural equation model or through multigroup analysis to examine potential subgroup differences.

Fifth, although we implemented procedural and statistical remedies to control for common method bias, and discriminant validity among the key constructs was statistically established, the cross-sectional, self-reported nature of the data means we cannot completely rule out its potential influence. The consistently strong correlations, while theoretically expected, may still reflect some degree of shared method variance or common rater effects. Future research could benefit from employing multi-wave, multi-source data to further disentangle method effects from substantive relationships and strengthen causal inferences.

Finally, while the present study focused on self-efficacy and academic engagement as mediating patterns, other psychological and contextual factors may also influence students’ EAP-related PAA. Future research could explore additional variables such as academic emotions, resilience, feedback literacy, or cross-cultural adaptation in internationalized learning environments. Investigating these roles may further enrich understanding of how teacher support contributes to students’ academic development in globalized higher education settings.

## 7 Conclusion

For students in engineering-focused, male-majority cohorts within Sino-foreign cooperative “4 + 0” dual-degree programs, this study investigated the relationship between teacher support and students’ EAP-related PAA. Drawing on SCT, the study examined the mediating roles of self-efficacy and academic engagement in explaining how teacher support influences students’ perceptions of their academic performance in EMI learning contexts.

The results revealed that teacher support positively predicts EAP-related PAA both directly and indirectly. In particular, self-efficacy and academic engagement serve as important mediating mechanisms linking teacher support to students’ self-perceived academic performance. Moreover, the findings identified a sequential pathway in which teacher support enhances self-efficacy, which subsequently promotes academic engagement and ultimately contributes to higher levels of EAP-related PAA in EMI context.

By highlighting the psychological and behavioral processes underlying this relationship, the present study, conducted with a sample of students in engineering-focused, male-majority cohorts, contributes to a deeper understanding of such students in specific internationalized higher education contexts (i.e., Sino-foreign “4 + 0” programs). The findings suggest that fostering supportive instructional environments that strengthen students’ efficacy beliefs and encourage active engagement may play a crucial role in promoting positive academic experiences in English-medium and cross-cultural learning settings.

Overall, this study underscores the importance of teacher support as a key contextual resource for enhancing students’ motivation, engagement, and perceived academic success of students within similar educational and demographic profiles in globalized higher education.

## Data Availability

The raw data supporting the conclusions of this article will be made available by the authors, without undue reservation.

## Footnotes

1
https://www.wjx.cn/
